# Dental Home Visits for Caries Prevention Among Preschool Children: Protocol for a Cost-Effectiveness Analysis on a Randomized Control Trial

**DOI:** 10.2196/10053

**Published:** 2018-06-06

**Authors:** Niekla Survia Andiesta, Maimunah A Hamid, KKC Lee, Allan Pau

**Affiliations:** ^1^ School of Dentistry International Medical University Kuala Lumpur Malaysia; ^2^ School of Medicine International Medical University Kuala Lumpur Malaysia; ^3^ School of Pharmacy Monash University Kuala Lumpur Malaysia

**Keywords:** cost-effectiveness analysis, dental home visits, caries prevention, preschool children

## Abstract

**Background:**

In 2012, nearly 4000 children in Malaysia were referred to hospital pediatric dental services due to dental caries. Recent research has reported the effectiveness of dental home visits in preventing caries development in young children. Dental home visits (DHVs) are described as an ongoing relationship between the dentist and their patients, providing all aspects of a preventive oral health care program in the presence of the parents at home.

**Objective:**

The objective of this study is to evaluate the cost-effectiveness of dental home visits and oral health information, in the form of educational leaflets, in preventing new caries development in young children, compared to those receiving only educational leaflets over a period of two years. Cost-effectiveness analysis will be used to evaluate the cost-effectiveness of dental home visits.

**Methods:**

This is a collaborative project with the Oral Health Division of the Ministry of Health Malaysia. The Oral Health Division will provide access to a subsample from the National Oral Health of Preschoolers Survey which was carried out in 2015. The population of interest is children aged 5 and 6 years from kindergartens in the Selangor state of Malaysia. The study adopted a societal perspective for cost-effectiveness analysis and all types of resources that are of value to society will be included in analyzing the costs; such as cost to the patient, cost to the provider or institution, and indirect costs because of loss of productivity.

**Results:**

The trial has been approved by the International Medical University Malaysia’s Joint Research and Ethics Committee (Project ID: IMU R157-2014 [File III – 2016]). This trial is currently recruiting participants.

**Conclusions:**

The number of young children in Malaysia who have been referred to the hospital children’s dentistry service for severe caries is disturbing. The cost of dental treatment in young children is high due to the severity of the caries which require an aggressive treatment, and the need for general anesthesia or sedation. This study will provide information on the cost and effectiveness of DHVs in caries prevention of young children in Malaysia.

**Registered Report Identifier:**

RR1-10.2196/10053

## Introduction

In Malaysia, 86% of rural and 69% of urban preschool children have experienced dental caries, most of which remain untreated [1]. The dental treatment cost in young children is high due to the need for treatment under general anesthesia or sedation, therefore causing significant economic and social burdens on the families [2,3]. In 2012, nearly 4000 children in Malaysia were referred to hospital pediatric dental services because of dental caries [4].

Recent research has reported the effectiveness of dental home visits (DHVs) in preventing the development of caries in young children. The American Academy of Pediatric Dentistry describes DHVs as an ongoing relationship between the dentist and their patient, providing all aspects of a preventive oral health care program in the presence of the parents at home [5,6]_._ This concept has been reported to result in a sustainable oral health behavioral change as the child is more likely to receive appropriate preventive and routine oral health care, thereby reducing the risk of preventable dental disease [7]. In addition, DHVs have the advantage of providing a personalized preventive intervention based on a child’s needs [8].

A study reported in 2013 by Plonka et al showed that oral health education for children through home visits significantly reduced the rate of development of early childhood caries (ECC) compared to those who were contacted via telephone [9]. The results of this study showed the benefit of personal contact in home visits for delivery of oral health education, and this significantly reduced the number of children who developed ECC [9].

Other studies have investigated the cost-effectiveness of DHVs in caries prevention. One such example is a study which investigated the benefit-cost and cost-effectiveness of a long term dental health program for prevention of ECC in infants through home visits, and this study showed favorable results [10]. In a separate study, a home-visit intervention for ECC was found to be more cost-effective than a telephone intervention delivery mode, contributing significant cost savings to the public health care system [11].

Educational leaflets (ELs) are one of the materials commonly used in oral health promotion programs, which reinforce oral health education messages and are frequently used for the benefit of dental patients to complement communication with the dentist [12,13].

Cost-effectiveness analysis (CEA) is widely used to inform decision-makers about the value of new health programs and interventions. CEA is the most commonly used technique for economic evaluation and it estimates the costs and effects of health interventions and sums up the results in a cost-effectiveness ratio [14].

Given the high prevalence of dental caries in Malaysia, and the availability of effective DHVs and ELs material to support oral health promotion, the rationale of this study is to identify effective and cost-effective behavioral interventions to promote oral health and reduce oral health disparities in the population [1,7,8,12,13]. Health behaviors and lifestyles can influence both the oral and general long-term health of the population which can have economic and social consequences [15]. Furthermore, this is the first reported study of DHVs in Malaysia and an economic evaluation in this area is needed to estimate the value of the outcomes received for the expenditures spent on this intervention.

The aim of this study is to evaluate the cost-effectiveness of DHVs and oral health information in the form of ELs in preventing new caries development in young children, compared to those receiving only ELs over a period of 2 years. The specific objectives are:

1. To determine the cost of DHVs and ELs for prevention of new caries per child compared to the cost for a child only receiving oral health information through ELs for prevention of new caries after a 2-year follow-up.
2. To determine the cost of DHVs and ELs in improving the oral health-related quality of life (OHRQoL) for one child compared to the cost of only ELs in improving the OHRQoL for one child after a 2-year follow-up.
3. To compare the cost of dental treatment incurred and all associated expenditures among children receiving DHVs and ELs and those receiving only ELs over 2 years.

## Methods

### Study Design

This is a CEA of DHVs to families of young children for caries prevention compared to young children receiving oral health information in the form of ELs over a period of two years. For the CEA, the study adopted a societal perspective, which has been suggested as the most appropriate and comprehensive perspective [14]. In this perspective, all types of resources that are of value to society will be included in analyzing the costs; such as cost to the patient, cost to the provider or institution, and indirect costs because of loss of productivity.

### Ethics, Consent, and Permission

Ethics approval for this project will be obtained from the International Medical University Malaysia’s Joint Research and Ethics Committee. Approval for cooperation from the selected kindergartens will be obtained from the Ministry of Education of Malaysia.

### Recruitment

This is a collaborative project with the Oral Health Division (OHD) of the Ministry of Health Malaysia. The OHD will provide access to a subsample population from the National Oral Health of Preschoolers Survey (NOHPS) which was carried out in 2015. The population of interest is children aged 5 and 6 years from kindergartens in the Selangor state in Malaysia. The names and addresses of the kindergartens will be obtained from the OHD.

Upon obtaining an appointment, the researchers will visit the selected kindergartens to provide details about project to the heads of the kindergartens and determine their interest in participating in the study. The heads will be asked to distribute the information leaflets and consent forms to all 5- and 6-year old children in the kindergarten to be taken home to their parents. The information leaflet will include details about the study as wells as the researchers’ names and telephone numbers for any further enquiries. Parents will be given a week to read the information leaflet and to allow parents time to make any enquiries necessary before giving consent. The parents who agree to participate in the study will be asked to sign the consent form and provide their phone numbers, addresses, and children’s names for future contact. The forms will be returned to the kindergarten. Participants in this study will be identified from the consenting families and only children aged 5 or 6 years at commencement of the study will be eligible for inclusion.

### Sample Size and Randomization

It is estimated that there will be a 5% of incidence of caries in the intervention group during the follow-up, compared with 20% in the control group. This is based on the findings of a study conducted in 2015 by Koh et al that reported a 4% of incidence of caries in the intervention group and 39% in the control group [11]. Using a statistical software, Epi info 7 (Center for Disease Control and Prevention, USA, 2015), a sample size calculation was conducted and a minimum sample of 176 subjects is needed to detect a 15% difference in incidence of caries, with a significance level of 95% and a power of 80% [16]. The researchers decided to aim for doubling the effective sample size to 300 subjects to ensure the effective sample size will be higher than 176 with a high power. After taking sampling error into account and the assumption of a 30% nonresponse rate, followed by the assumption of another 30% of drop-out rate, a total of 600 subjects will be approached to participate in this study.

The kindergartens that agreed to participate will be randomly allocated into either the intervention or control group (1:1) by a random “draw” method. No matching will be done since they all have a similar socioeconomic background. All children aged 5 or 6 years at the participating kindergartens, with parents’ consent to participate, will be included in the study [17].

### The Intervention

All consenting families in the intervention group will receive DHVs and ELs delivering dental care advice by a team of two dental home visitors at 6-month intervals. This advice will include information regarding daily tooth brushing, simple diet advice to prevent caries, and information on the need for regular dental check-ups. The families will also be provided with information about the dental services available in their vicinity and how to access these services. The duration of each visit will be approximately 30 minutes. A personalized approach that avoids direct persuasion will be used, and the dental team will acknowledge the parents’ right to choose when providing feedback and advice about dental care [18].

### Control Group

The control group will receive ELs that will be delivered by hand at 6-month intervals for the 2-year study period by the dental home visitors.

### Study Measures

#### Outcome Measures

The primary outcome of this study is the incidence of new caries, measured by the number of children included in the study with new caries, over a period of 2 years. The difference in the number of children with new caries between the intervention and the control groups will be considered as the number of children prevented from developing caries.

The calculated cost of the DHVs used in the intervention will include staff salaries, telephone calls to make appointments, travelling allowances, and administrative costs. The ELs cost is estimated by using the market price to purchase leaflets, staff salaries, telephone calls, travelling allowances, and administration costs. The cost of preventing a child from developing caries will be calculated as the DHVs and ELs cost divided by the number of children prevented from new caries.

The secondary outcome of this study will be the improvement in OHRQoL over a period of 2 years. This is measured by the improvement in the early childhood oral health impact scale (ECOHIS) scores. The difference in the ECOHIS scores between the intervention and the control groups will be considered as the improvement in OHRQoL. The cost of improving the OHRQoL is calculated as the DHVs and ELs cost divided by the improvement in the ECOHIS scores.

#### Number of New Carious Teeth

An oral examination will be conducted at baseline (before the start of the intervention) and at the end of the 2-year study period by researchers using the NOHPS protocol, which was based on the World Health Organization (WHO) recommendation published in 2013 [19]. The number of new decayed, missing (due to caries), and filled posterior deciduous teeth (dmft), as well as the number of decayed, missing (due to caries), and filled first permanent molars (DMFT) will be recorded. The dmft and DMFT caries assessment is chosen to ensure consistency with the assessment used by the OHD Ministry of Health Malaysia.

#### Oral Health-Related Quality of Life Improvement

The OHRQoL improvement will be measured using the ECOHIS questionnaire. The ECOHIS questionnaire is a specific instrument developed by Pahel et al in 2007 to assess the perception of parents on OHRQoL of preschool children. It has 13 items, distributed between 2 sections, namely the Child Impact Section and the Family Impact Section. The Child Impact Section has 4 domains (child symptom, function, psychological, and self-image or social interaction); and the Family Impact Section has 2 domains (parent distress and family function). Responses are recorded using a 5-point Likert scale to record how often an event has occurred where 0=never, 1=hardly ever, 2=occasionally, 3=often, 4=very often, and 5=don’t know [20]. The ECOHIS has been validated on a Malaysian population and was found to have high validity (Cronbach alpha=.83) and high reliability (kappa=.95) in assessing the impact of oral disorders or conditions on the quality of life of urban preschool children age 4 to 6 years old as reported by Hashim et al [21].

The ECOHIS questionnaire will be completed by the parents in both the intervention and control groups at baseline and at the end of the 2-year follow-up period. Based on the responses, the ECHOIS score as prescribed by Hashim will be computed. The ECOHIS score at the end of the 2-year follow-up will be used to assess the improvement of OHRQoL of the children in both intervention and control groups.

#### Costs of Dental Home Visits and Educational Leaflets

The DHVs cost data will include the cost of dental home visitors, telephone calls, administration costs, and travelling costs of dental home visitors. The costs of the dental home visitors will be calculated according to the average salary of a dental nurse; the costs of the telephone calls used to make appointments for DHVs will be estimated according to the fees of prepaid phone number; and the travelling costs of the dental home visitors will be estimated by the average travel allowance per day in the Selangor area. The administration cost will include the costs of paper, stationary, and photocopies used for the informed consent forms and letters to authorities, as well as the training cost for delivering intervention. As the ELs used in the study will be obtained from existing Ministry of Health leaflets which are most relevant to the study objectives, the ELs cost in this study will be estimated by using the market price for purchase of leaflets, staff salaries, telephone calls, travelling allowances, and administration costs.

#### Costs of Dental Treatment

The cost data for dental treatment incurred by the families will be obtained from the recorded dental visit diary completed by the parents. The dental visit diary will be created to record every dental visit made during the study period and checked by the researchers every six months until the end of the study period. The information recorded in the diary will capture the reason for the dental visit, the type of dental clinic visited, the dental treatment or procedure conducted, the time spent at the dental clinic, the time spent travelling to and from the dental clinic, the total cost of the dental treatment or procedure, the cost of travelling to and from the dental clinic, the person who brought the child to the dental clinic, and the number of parents’ days off used to bring the child to the dental clinic. The use of dental visit diary has been reported to be suitable for self-completion by the parents [22].

The dental visit diary will be created according to an annotated cost questionnaire as proposed and validated by Thompson and Wordsworth for the UK Health Economics Research Unit [22]. It will be translated to Bahasa Malaysia using the forward and backward translation process. It will be pretested to assess its relevance and ease of understanding by the potential participants in the context of the communities included in the study.

### Data Collection Procedure

#### Standardization

Standardization and calibration training will be carried out by the OHD Ministry of Health Malaysia for the researchers in this project to ensure the reliability of the measurements in oral examination at the baseline and at the end of the 2-year follow-up period. The training is carried out in order to achieve the following outcomes:

1. Uniform interpretation, understanding, and application of recording instructions and criteria.
2. Standardization and calibration of chosen examiners (researchers) against the benchmark examiner (OHD personnel).
3. Reasonable consistency with minimal inter- and intraexaminer variability.
4. Familiarization with survey forms, field procedures, and equipment to be used [23].

#### Baseline Data

The baseline clinical data for all study participants will be based on the oral examination to be carried out at the kindergarten for all children with the parents’ consent. This examination will be conducted by researchers who have successfully attended the standardization training session. Oral examination will use a disposable mouth mirror. Caries assessment will be based on visual inspection, without invasive probing, and is diagnosed at the cavitation stage. Both primary and permanent teeth will be examined and if a permanent and a primary tooth occupy the same space, then only the status of the permanent tooth will be recorded. Caries assessment will adhere closely to the NOHPS criteria [17], which has been modified in 2013 from the WHO recommendations [21]. It is important to note that the WHO uses alphabets to denote caries status for primary teeth, however, NOHPS protocol uses numeral for both primary and permanent teeth. Hence, in this study, the following coding will be used: Sound teeth=0 (instead of A), Caries=1 (instead of B), Filled with caries=2 (instead of C) and Filled no caries=3 (instead of D). The teeth examination will begin from upper right to upper left and lower left to lower right quadrant and a tooth will be considered present when any part of it is visible [19,23]. No radiographs will be taken, and no treatment will be provided. Children who have been assessed with obvious caries will be advised to visit a dental clinic for further assessment and for treatment if necessary.

Baseline DHVs will be carried out and the parents in both the intervention and control groups will receive the ECOHIS questionnaire and a dental visit diary. The baseline ECOHIS questionnaire will be filled by the parents during the visit and returned to the researchers. The dental visit diary will be maintained by the parents and the research team will check the diary every 6 months. Parents will be informed to complete the diary every time their children visit the dental clinic.

#### Follow-Up 6-Monthly Visits

Follow-up visits will be made every 6 months at the 6th, 12th, 18th month of the study period to the homes of both the groups. The parents in intervention group will receive DHVs and ELs by the dental home visitors, while those in the control group will receive only ELs to be delivered by the dental home visitors. The researchers will check the dental visit diary from both groups at each 6-month visit.

#### The 2-Year Follow-Up Data

At the end of the 2-year study period, the researchers will visit the subjects’ homes from both the intervention and control groups and repeat the same dental examination, complete the ECOHIS questionnaire, and gather the information from the dental visit diary. At the 2-year follow-up visit, the subjects will be 7 or 8 years old. It is anticipated that, should new caries develop, the majority will be on primary posterior teeth and first permanent molars. Adhering to the NOHPS protocol, the follow-up oral examination will be carried out to collect the number of new decayed, missing (due to caries) and filled posterior deciduous teeth (dmft), and the number of decayed, missing (due to caries) and filled first permanent molars (DMFT). The children will be examined while sitting on a portable dental chair at their homes. The visual oral examination will use disposable mouth mirrors with natural daylight for illumination, supported by a Daray lamp where necessary. No radiographs will be taken, and no treatment will be provided. Children who have been assessed with obvious caries will be advised to visit a dental clinic for further assessment and treatment if necessary. The researcher will offer to provide information or make the necessary arrangements for families who wish to have further assessment at a dental clinic.

### Data Analysis

Data collected from each visit will be checked for completeness by the research team members before it is keyed into SPSS. All the outcomes measures will be analyzed using the SPSS Program. Descriptive statistics will be used to report new caries teeth, OHRQoL, cost of DHVs and ELs, and the incremental cost effectiveness ratio (ICER) at 6, 12, and 18 months, as well as at the end of the 2-year follow-up period. A *t* test will be used for comparison of means of those parameters between the two groups and over the period of study. Logistic regression analysis will be carried out to test the influence of individual characteristics and on the CEA of the intervention in preventing caries.

### Cost-Effectiveness Analysis

The CEA will measure cost in monetary units of DHVs and ELs. The effectiveness of the study is measure by the outcomes in number of teeth saved and the ECOHIS score at the 2-year follow-up. CEA assesses the cost of the study through the use of the ICER [8,24,25] and is defined as the ratio of the difference in costs divided by the difference in outcomes. The ICER for this study will be generated by calculating the difference in cost for dental treatment between the intervention group and the control group, divided by the difference in outcomes between the intervention group and the control group (ie, the number of new carious teeth and ECOHIS scores) [8,24,25]. Based on the data collected, the outcome measure for OHRQoL is only from the ECOHIS questionnaire, therefore this study focuses on CEA as a procedure to decide the most cost-effective way to achieve the objectives [11]. Discounting and sensitivity analysis are not conducted because the study does not perform any projection of future years and the cost data of this study is the real-world cost data [14].

## Results

The trial has been approved by the International Medical University Malaysia’s Joint Research and Ethics Committee (Project ID: IMU R157-2014 [File III – 2016]). Participants are currently being recruited for inclusion in the study.

## Discussion

The cost of dental treatment in young children is high due to the severity of the caries developed which can require an aggressive treatment, as well as the need for general anesthesia or sedation. Children who receive treatment under general anesthesia frequently require further hospitalization for new lesions, some as soon as 6 months after the first general anesthesia. Clearly, existing health services need to be supplemented with a population-based approach to promote child oral health. This is the first study of this intervention in Malaysia, and economic evaluation in this area is crucial to estimate the value of the outcomes received for the expenditures spent on this intervention. This study will provide information on the cost and effectiveness of DHVs in caries prevention of young children in Malaysia.

## Abbreviations

CEA: cost-effectiveness analysis
dfmt: decayed, missing (due to caries), and filled posterior deciduous teeth
DFMT: decayed, missing (due to caries), and filled first permanent molars
DHV: dental home visit
ECC: early childhood caries
ECOHIS: early childhood oral health impact scale
EL: educational leaflet
ICER: incremental cost-effectiveness ratio
NOHPS: National Oral Health of Preschoolers Survey
OHD: Oral Health Division
OHRQoL: oral health-related quality of life
WHO: World Health Organization
